# *A20* inhibits doxorubicin-induced macrophage maturation and apoptosis through mTOR signaling in classical Hodgkin lymphoma

**DOI:** 10.22038/ijbms.2025.86862.18767

**Published:** 2025

**Authors:** Nguyen Xuan Canh, Phan Thi Hoai Trang, Pham Thi Huong, Do Thi Trang, Pham Viet Nhat, Nguyen Tien Manh, Lê Duy Thành, Nguyen Trung Nam, Nguyen Ba Vuong, Nguyen Thi Xuan

**Affiliations:** 1 Faculty of Biotechnology, Vietnam National University of Agriculture, Hanoi, Vietnam; 2 Military Hospital 103, Vietnam Military Medical University, Phung Hung, Ha Dong, Hanoi, Vietnam; 3 Department of Pathophysiology, Vietnam Military Medical University, Phung Hung, Ha Dong, Hanoi, Vietnam; 4 Institute of Biology, Vietnam Academy of Science and Technology, 18 Hoang Quoc Viet, Cau Giay, Hanoi, Vietnam; 5 Publishing House for Science Technology, Vietnam Academy of Science and Technology, 18 Hoang Quoc Viet, Cau Giay, HaNoi, Vietnam; 6 Department of Military Epidemiology, Vietnam Military Medical University, Phung Hung, Ha Dong, Hanoi, Vietnam; 7 108 Military Central Hospital, Tran Hung Dao, Hai Ba Trung, Hanoi, Vietnam

**Keywords:** A20, Classical hodgkin lymphoma, Everolimus, IL-1β, Macrophages

## Abstract

**Objective(s):**

Classical Hodgkin lymphoma (cHL) is identified by the appearance of Hodgkin and Reed-Sternberg cells. *A20* and *CYLD* are deubiquitinating enzymes involved in negatively regulating NF-κB-mediated immune response. Vincristine (Vinc) and doxorubicin (Dox) are classical antitumor drugs, in which Dox serves a key role in chemotherapy against cHL and Vinc induces disruption of microtubule function that inhibits mitosis of cancer cells. Little is known about the roles of *A20/CYLD* in regulating macrophage function from cHL patients upon treatment with Vinc or Dox. This study, therefore, asked whether *A20/CYLD* expression affects function of macrophages in cHL cases.

**Materials and Methods:**

Macrophages from cHL patients differentiated from bone marrow cells were exposed to Vinc or Dox. Gene expression levels were determined by real time-qPCR, cell maturation, apoptosis and phagocytosis by flow cytometry, and cytokine release by ELISA.

**Results:**

Dox induced maturation, apoptosis, and phagocytosis of macrophages in cHL cases. Moreover, the percentage of CD68^+^CD40^+^, but not CD68^+^CD86^+^ cells as well as levels of IL-1β were further enhanced when exposed to *A20* siRNA, whereas the absence of *CYLD* unaltered macrophage function in cHL patients. Importantly, the increased numbers of *A20*-sensitive CD68^+^CD40^+^ and Annexin V^-^PI^+^ cells as well as enhanced levels of caspase 3 were abolished in the presence of mTOR inhibitor Everolimus.

**Conclusion:**

The present study indicates that Dox-induced macrophage maturation and apoptosis are dependent on *A20* expression through mTOR signaling. Moreover, inhibition of Dox-induced macrophage maturation in the patients with low A20 expression by Everolimus might represent a promising therapy for *A20*-sensitive cHL cases.

## Introduction

Classical Hodgkin lymphoma (cHL) is identified by rare Hodgkin and Reed-Sternberg cells (HRS) surrounded by reactive immune cells, including macrophages (1, 2). Although cHL is a highly curable lymphoid malignancy by modern treatments, about 20% of patients still have relapsed/refractory disease (3). HRS cells are positive with CD30, a cell membrane protein and a more variable expression pattern of CD15 in approximately 70% of cases (1). Differently, CD20^ +^ cells display a favorable outcome in cHL (4). HRS cells of cHL originate from the germinal centre B cells, however, the expression of classic B lineage markers is lost on their cell membrane (5). cHL has been classified into four histological subgroups: nodular sclerosis, mixed cellularity, lymphocyte-rich and lymphocyte-depleted cHL (1). Recent research has indicated that single mutations, and several aberrant signaling pathways involved in blocking recruitment of natural killer (NK) and cytotoxic T cells, including the programmed cell death ligand (PD-L)1/PD-1, nuclear factor-kappa (NF-κ)B, and Janus kinase - signal transducer and activator of transcription (JAK/STAT) pathways promote survival and proliferation of HRS cells as well as evade antitumor immune mechanism in cHL (1, 5). 

HRS cells secrete macrophage colony-stimulating factor (M-CSF) to induce macrophage differentiation from monocytes (6), therefore, increased infiltration of CD68^+^ macrophages correlate with a poor prognosis in cHL (7). Macrophages are an important component of the innate immune response by ingesting and eliminating foreign substances and circulating cancer cells (8). The phagocytosis of them leads to antigen presentation and induces the anti-tumor immune responses (9). An increase of CD68^+^CD163^+^ tumor-associated macrophages (TAM) is considered an unfavorable prognostic factor in patients with B-cell lymphoma (10). Moreover, the proliferation and non-specific activation of macrophages in HL lead to macrophagic activation syndrome, which is characterized by an excessive, and uncontrolled immune response and rapidly fatal if it is not managed (8). Therefore, macrophages are selected as target cells in this study.

*A20*, also called tumor necrosis factor alpha induced protein 3 (TNFAIP3) and tumor suppressor cylindromatosis (*CYLD*) are deubiquitinating enzymes involved in negatively regulating NF-κB-mediated immune response through expression of proinflammatory and survival genes (11-13). *A20* gene mutations are detected in cHL (14). Expression of *A20* is inactivated in multiple types of B-cell lymphoma (15). Mice lacking A20 die prematurely by excessive multi-organ inflammation. A20 inhibits inflammatory response and apoptosis in macrophages (16). Besides, A20 suppresses activation of NK cells (17) and promotes survival of CD4 T cells (18) through the mammalian target of rapamycin (mTOR) signaling. Unlike *A20*, levels of *CYLD* are down-regulated in patients with leukemia (12). CYLD induces the death of hepatocellular carcinoma (HCC) cell lines when treated with doxorubicin through NF-κB activation (13). Our recent study indicates that CYLD inhibits maturation and promotes apoptosis and phagocytosis of macrophages in patients with acute myeloid leukemia (12). CYLD is known to regulate cognitive function, hippocampal plasticity and autophagy via mTOR signaling (19) and suppresses Akt and mTOR activity and promotes autophagy at the synapse (20). In mice, *CYLD*-knockout mice exhibit abnormalities in the activation and development of immune cells (21).

Vincristine (Vinc) and doxorubicin (Dox) are classical antitumor drugs, in which Dox serves a key role in chemotherapy against cHL. The primary cytotoxic mechanism of Vinc involves interaction with tubulin and subsequent disruption of microtubule function that primarily inhibits mitosis of cancer cells and frequently triggers neuropathic pain (22). Unlike Vinc, Dox induces immunogenic cell death, which stimulates the induction of immunostimulatory properties, resulting in an adaptive immune response (23). Dox inhibits DNA replication and transcription by DNA intercalators and prevents activation of topoisomerase II, leading to DNA fragmentation and cell death (23). Vinc-induced cell apoptosis is detected at of concentration of 2-50 nM (24), while Dox shows cytotoxicity at a concentration of 0.1–50 μM (25). The effects of Dox and Vinc in promoting the release of IL-1β by bone marrow-derived macrophages are shown in a recent study (26). Treatment of antigen-presenting cells with Dox enhances the phagocytosis of cancer cells (27).

The role of *A20/CYLD* in the modulation of macrophage function in cHL is little known. Activation of which is associated with a high risk of macrophagic activation syndrome and uncontrolled immune response in these patients, therefore, we asked whether *A20/CYLD* affect biological functions of macrophages upon treatment with Vinc or Dox. To this end, macrophages differentiated from bone marrow cells of cHL patients were exposed to Vinc or Dox and cytokine production, maturation and apoptotic cell death were examined. Expressions of signaling molecules, including mTOR, mitogen activated protein kinase (MAPK)p38, extracellular signal-regulated kinase (ERK), Wnt and AKT were assessed in Vinc/Dox-treated macrophages to determine the mechanism involved in *A20*-sensitive macrophage activation in cHL patients.

## Materials and Methods

***Patient subjects***

Fifty-eight diagnosed cHL patients participated in the study at the National Institute of Hematology and Blood Transfusion, Vietnam National Cancer Hospital, 108 Military Central Hospital, and 103 Military Hospital, Ha Noi, Vietnam. The 2016 World Health Organization (WHO) criteria (28) was used for diagnosis of cHL. The control population did not take any medication or suffer from any known acute or chronic disease. All volunteers gave a written consent to participate in the study. The study protocol was approved by the Ethical Committee of Institute of Genome Research. 

***Generation of Human macrophages***

Bone marrow (BM) cells from cHL patients and venous blood from healthy volunteers were collected into sterile EDTA tubes. The density gradient centrifugation (Ficoll Histopaque, Cytiva, 17144003) was used to isolate BM and peripheral blood mononuclear cells (PBMCs). Freshly isolated BM cells and PBMCs were harvested by centrifuging at 400g for 30 min. The cells were washed with phosphate buffered saline (PBS, Thermo Fisher Scientific, 10010023), and cultured in RPMI 1640 medium (Thermo Fisher Scientific, 11875093) with 10% fetal bovine serum (FBS, Thermo Fisher Scientific, 10270106), L-glutamine (Thermo Fisher Scientific, A2916801), Penicillin-Streptomycin (Thermo Fisher Scientific, 15140122), and MEM non-essential amino acids (NEAA, Sigma Aldrich, M7145). Differentiation of macrophages from BM cells or PBMCs was achieved by using M-CSF (50 ng/ml, Peprotech, 300-25) for seven days. At days 3 and 5, cultures were fed fresh medium and M-CSF. At day 8, cells were treated with siRNA control (40nM, Thermo Fisher Scientific, 4390844) or siRNA *A20* (40nM, Thermo Fisher Scientific, 121590) or siRNA *CYLD* (40nM, Thermo Fisher Scientific, 4390825) for 48 hr with or without of Vinc (150nM, Abcam, ab120226) or Dox (5 µM, Abcam, ab120629) for another two hours.

***Preparation of macrophages with siRNA***

Control- or *A20* or *CYLD*-targeted siRNA was added to macrophage culture (2 x 10^5^ cells/1 ml) in combination with Lipofectamine***® ***3000 ***(***Thermo Fisher Scientific, L3000008). Cells were treated with the siRNAs for 48 hr at 37^0^C, 5% CO_2_. Cells were the exposed to Vinc or Dox for further experiments.

***Immunophenotyping***

The expression levels of CD86 (Thermo Fisher Scientific, 12-0869-42), CD40 (Thermo Fisher Scientific, MA1-10222), and CD68 (Thermo Fisher Scientific, MA523616) were determined by flow cytometry. Cells (2 x 10^5^) were incubated with fluorochrome-conjugated antibodies at a concentration of 10 µg/ml. After incubating with the antibodies for 60 min at 4 °C, the cells were washed twice and resuspended in FACS buffer for flow cytometry analysis (FACSAria Fusion, BD Biosciences).

***Measurement of cytokine secretion***

Macrophages were cultured with control or *A20* or *CYLD* siRNA and then added with Vinc (150 nM) or Dox (5 µM). Cell culture supernatant was harvested and stored at -20 °C. Interleukin (IL)-1β concentrations were measured by using ELISA kit (Thermo Fisher Scientific, BMS224INST) according to the manufacturer’s protocol. 

***Quantitative polymerase chain reaction (qPCR)***

The isolation of RNA was carried out using the Qiashredder and RNeasy Mini Kit (Qiagen, 69504) according to manufacturer’s instructions. cDNA was synthesized from mRNA and determined the transcript levels of *A20, CYLD,* *mTOR*, *MAPKp38, ERK2 Wnt1, Wnt3*,* AKT1, Bax*, *caspase 3 *and *caspase 8* by using the LightCycler System (Roche Diagnostics). The qPCR primer sequences of the genes are listed in Table 1. 

Quantitative PCR reactions were performed under the following conditions: 40 cycles of 95 °C for 10 sec, 62 °C for 10 sec, and 72 °C for 16 sec, each with a temperature transition rate of 20 °C/sec, a secondary target temperature of 50 °C, and a step size of 0.5 °C. Melting curve analysis was performed at 95 °C, 0 sec; 60 °C, 10 sec; 95 °C, 0 sec to determine the melting temperature of primer dimers and the specific PCR products. The relative quantification of the genes was calculated according to the 2^-ΔΔCT^ method by using corresponding glyceraldehyde 3-phosphate dehydrogenase (GAPDH) gene.

***Phagocytosis of macrophages***

Macrophages were cultured for three hours with carboxyfluorescein diacetate succinimidyl ester (CFSE, Thermo Fisher Scientific, C34554) to attain CFSE-labeled lymphoma cells. These cells were mixed with macrophages at the ratio of 1:2 and then incubated with anti-CD68 for one hour at 4 °C for flow cytometry. The CD68^+^CFSE^+^ cells were determined as phagocytosing cells.

***Apoptosis assay***

Macrophages were stained with allophycocyanin (APC)-conjugated annexin V and propidium iodide (PI) (Thermo Fisher Scientific, 88-8006-74) in the dark for 15 min at room temperature. The cells were then washed with Annexin washing buffer (AWB) and analysed by flow cytometry. 

***Statistical analysis***

Statistical analyses were performed using SPSS version 20 (IBM, New York, NY, USA) and GraphPad Prism version 8.4 (GraphPad Software, San Diego, CA, USA). Differences were tested for significance using the Mann–Whitney U test. Statistical significance was set at *P*<0.05.

## Results

***Association between A20/CYLD gene expression and clinical features in cHL***

Fifty-eight patients with cHL were enrolled and clinical association with *A20* and *CYLD* expressions are shown in Table 2. Firstly, the expression levels of *A20*/*CYLD* consisted of two groups based on the median of their values in healthy controls (high vs low). The high *A20* expression group was found in 40 samples (68.97%) and the low *A20* expression group was observed in 18 samples (31.03%​). The high *CYLD* expression group was seen in 44 samples (75.86%) and the low *CYLD* expression group was observed in 14 samples (24.14%) (Table 2). Our data showed that patients with the low *A20* expression had significantly higher levels of total protein and ALT, whereas patients with the high *CYLD *expression had significantly higher levels of total protein and creatinine. In addition, no association among other clinical indicators with *A20 *and *CYLD* expression levels was found (Table 2). 

***Regulatory effects of A20 on Doxorubicin-induced macrophage maturation and apoptosis in cHL patients***

Since activations of *A20* and *CYLD* were linked to changes in clinical features in cHL patients, therefore, we asked whether macrophage function in cHL patients is affected by the presence of these genes. Accordingly, macrophages from cHL patients were differentiated from bone marrow cells by M-CSF. At day 8 of the cultures, macrophages were added with either Vinc (50, 150 and 300 nM) or Dox (1, 5 and 10 µM) for two hours. The expression levels of *mTOR, MAPKp38, ERK2, Bax*, *Wnt1, Wnt3, AKT1,* *caspase 3, *and *caspase 8* were determined by real time quantitative PCR. The percentages of CD86 or CD40 positive and apoptotic/necrotic cells were examined by flow cytometry. The expressions of IL-1β, IL-6 and TNF-α were examined by ELISA.

**Figure 1:**
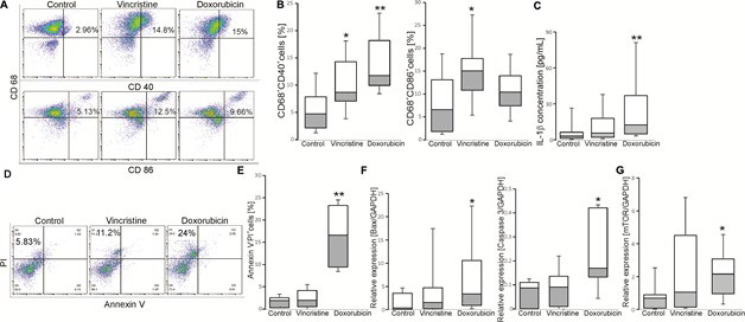
Effects of vincristine and doxorubicin on macrophage maturation and apoptosis in cHL patients

Firstly, we observed that the effects of maturation, apoptosis and cytokine secretion reached statistical significances at 150 nM Vinc and 5 µM Dox (Figure 1). Therefore, we used Vinc and Dox at concentrations of 150 nM and 5 µM, respectively for further experiments.

As shown in Figure 1A-B, CD68^+^gated cells were considered as macrophages. Results indicated that the number of CD68^+^CD40^+ ^cells was increased in the presence of Vinc or Dox, while the number of CD68^+^CD86^+ ^cells was enhanced in the presence of Vinc only. Moreover, the levels of cytokine IL-1β, but not IL-6 and TNF-α were increased when the cells were treated with Dox, but not Vinc (Figure 1C). 

Macrophage maturation leads to their apoptosis (29). Hence, we asked whether Vinc or Dox influences macrophage apoptosis from cHL patients. The number of apoptotic/necrotic (Annexin V^+^PI^-^ or Annexin V^+^PI^+^ or Annexin V^-^PI^+^) cells and the expressions of *Bax, caspase 3* and *caspase 8* were determined. As expected, treatment of macrophages with Dox, but not Vinc significantly enhanced the number of Annexin V^-^PI^+^(Figure 1D-E) cells and levels of *Bax* and *caspase 3, *but not* caspase 8* (Figure 1F). 

To examine whether several signaling molecules are related to the regulatory effects of Dox or Vinc, the expression levels of *mTOR, MAPKp38, ERK2, Wnt1, Wnt3,* and* AKT1 *were determined. As shown in Figure 1G, the levels of *mTOR, *but not *MAPKp38, ERK2, Wnt1, Wnt3* and* AKT1 *were enhanced when macrophages were treated with Dox.

**Figure 2:**
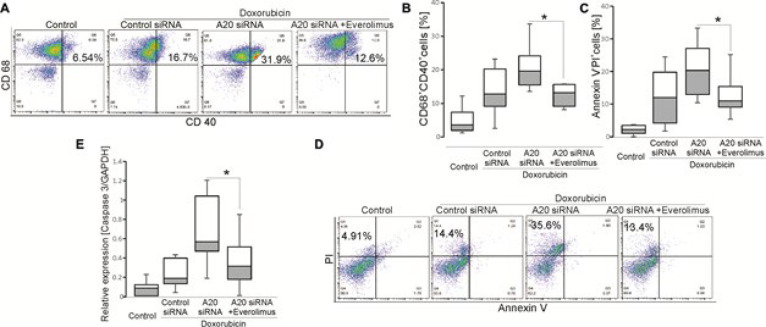
Roles of A20 on doxorubicin-induced macrophage maturation and apoptosis in cHL patients

Next, we asked whether the *A20* and *CYLD* influence macrophage functions when treated with Dox or Vinc. Results showed that the percentage of CD68^+^CD40^+^, but not CD68^+^CD86^+ ^cells as well as the levels of IL-1β were further enhanced when exposed to *A20* siRNA, whereas the absence of *CYLD* unaltered macrophage maturation in cHL patients (Figure 2A-C). Similar to maturation, macrophage apoptosis was affected by the absence of *A20*, as the percentage of Annexin V^-^PI^+ ^(Figure 2D-E) cells and levels of *Bax* and *caspase 3* (Figure 2F) were significantly elevated when the cells were silenced with *A20*, but not *CYLD*.

Finally, the expression levels of *mTOR* were examined. We observed that the mRNA levels of *mTOR* were significantly enhanced in the absence of *A20* (Figure 2G). In addition, mRNA levels of *MAPKp38, ERK2, Wnt1, Wnt3* and* AKT1 *were unaltered when cells were added *A20* siRNA or *CYLD* siRNA (data not shown). The evidences suggested that levels of *A20* were linked to the regulatory role of Dox on the maturation and apoptosis through mTOR signaling in macrophages from cHL patients.

***TmTOR signaling regulates A20-sensitive macrophage maturation and apoptosis upon Doxorubicin treatment in cHL patients***

**Figure 3:**
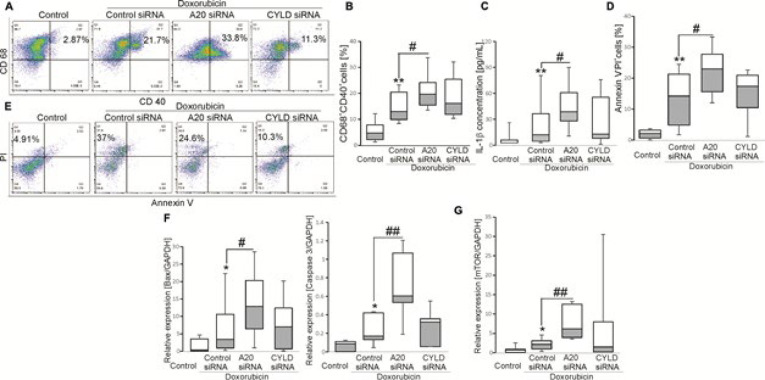
Effects of mTOR signaling on A20-sensitive doxorubicin-induced macrophage maturation and apoptosis in cHL patients

The mTOR activity is increased in patients with HL (2). To ask whether macrophage function from cHL patients is regulated by *A20* through mTOR signaling, cells were treated with Dox in combination with mTOR signaling inhibitor Everolimus with or without of *A20* siRNA. Importantly, the increase in the number of CD68^+^CD40^+ ^cells was abolished when cells were treated with Everolimus (Figure 3A-B). Besides, the cell death was rescued by addition of Everolimus, as the number of Annexin V^-^PI^+ ^(Figure 3C-D) cells and levels of *caspase 3* (Figure 3E) were unchanged when exposed to *A20* siRNA in the presence of Everolimus.

***Effect of doxorubicin on phagocytosis of lymphoma cells by macrophages from cHL patients***

**Figure 4:**
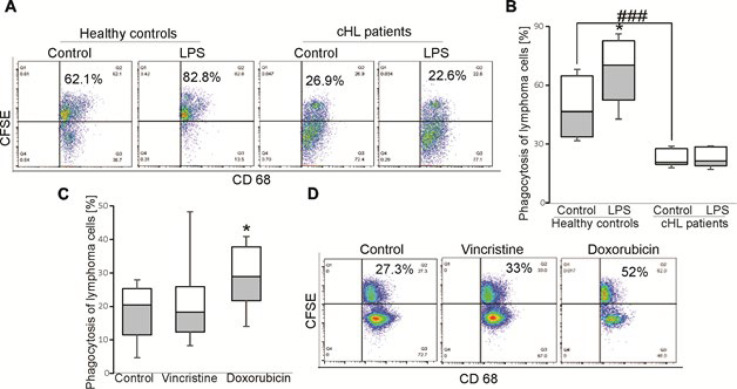
Effects of vincristine and doxorubicin on macrophage uptake of lymphoma cells from cHL patients

As shown in Figure 4A-B, macrophages from cHL patients phagocytosing lymphoma cells significantly less than healthy donors. In addition, LPS-stimulated macrophages had a significantly higher capacity to take up lymphoma cells than control cells, while phagocytosis of macrophages from cHL patients was unaltered when stimulated with LPS, suggesting that this function was affected in macrophages from cHL patients. Moreover, treatment of macrophages with Dox, but not Vinc enhanced the phagocytosis of lymphoma cells (Figure 4C-D). Nevertheless, the regulatory effects of *A20* and mTOR signaling on uptake of lymphoma cells by macrophages from cHL patients was not observed in this study (data not shown).

## Discussion

In this finding, cHL cases with the low levels of *A20* were associated with liver damage, as the elevation of total protein, and ALT levels in these patients. Differently, cHL cases with the high *CYLD *expression displayed significantly higher concentrations of total protein, and creatinine. The effects of *A20* and *CYLD* on regulation of cancer cell function are distinct. In our recent studies, *A20* promotes the uptake of lymphoma cells by dendritic cells from non-Hodgkin lymphoma (30), whereas *CYLD *stimulates macrophage phagocytosis in acute myeloid leukemia (12). The evidences suggest the effects of *CYLD* in negatively regulating functions of leukemic cells rather that lymphoma cells.

Moreover, the regulatory role of *A20, *but not* CYLD* was significantly related to Dox-treated macrophage maturation and apoptosis in cHL patients, as elevated expressions of the apoptotic markers, costimulatory molecule CD40 and levels of IL-1β in macrophages when exposed to Dox in the absence of *A20*, which was documented for the first time. Unlike Dox, Vinc at concentration of 150nM influenced CD86 expression on macrophages in cHL patients only. In agreement, the effects of Dox or Vinc on functions of macrophages were different from each other. The increased levels of Dox-induced IL-6 and MCP-1 are positively associated with macrophage apoptosis in mice (31). The high levels of CD86 are found in leukemia and leads to inactivation of T cells and relapse risk (32), whereas, expression of CD40 has diverse effects on cell functions to induce immune response (33). The enhanced expressions of CD40 and CD86 are known as M1-polarized macrophages, while the expression level of CD163 is highly observed in M2-polarized macrophages (33). Moreover, an increase in the number of M2 macrophages is linked to poorer overall survival in lymphomas patients (34). Differently, the promoting role of Vinc at concentration of ≥ 2nM on apoptosis of lymphoma cells are recently shown (24). Clearly, effects of Vinc and Dox on biological features are different from each other. Treatment with Vinc promotes neurotoxicity, while hematologic toxicity is rarely associated with Vinc (35). Vinc attenuates even Dox-induced cardiotoxicity (25). The effect of Dox on immunogenic apoptosis is well documented (31).

This study is in agreement with other investigations indicating that *A20* promotes the cell death in lymphoma cells (15) and suppresses cell proliferation and metastasis (36). In contrast, *A20* is known to negatively regulate cell death in glioblastoma, hepatocellular carcinoma, and acute lymphocytic leukemia (37). Clearly, the regulating roles of *A20* and *CYLD* on functions of macrophages are different. The expression levels of A20 are negatively associated with macrophage apoptosis (16), whereas CYLD stimulates macrophage apoptosis in acute myeloid leukemia (12). In agreement, A20 inhibits cell death in NK cells (17) as well CD4 T cells (18) through regulation of mTOR activity. Differently*, CYLD* did not affect activations of macrophages from cHL patients. *CYLD* participates in negatively regulating hyperresponsive inflammation in mouse macrophages (7). Inactivation of *CYLD* leads to a poor prognosis in marginal zone lymphoma (38), whereas, increased expression of CYLD is found in adult T-cell lymphoma (39). 

Moreover, the expression levels of *mTOR, *but not* Wnt, MAPK *and* AKT* signaling were enhanced in the presence of Dox without or with *A20* siRNA, but not CYLD siRNA. Therefore, blockade of mTOR signaling by Everolimus was used to examine whether maturation, apoptosis and the release of IL-1β by *A20*-dependent macrophages in cHL patients are associated with this signaling. The results indicated that mTOR signalinginhibitor Everolimus abolished the inhibitory roles of *A20* on macrophage maturation and apoptosis, but not IL-1β production, indicating that *A20* inhibited Dox-induced macrophage maturation and apoptosis via the mTOR pathway. MTOR signalingpathway is well known to play a key role in the development of lymphomas. It regulates cell cycle arrest, apoptosis and autophagy of lymphoma cells (40). Its expression is activated in 93% of HL cases (41) and the mTOR inhibitor Everolimus is considered an antiproliferative agent as well as targeted treatment for refractory/relapsed HL (2), while a combination of Akt and mTOR inhibitors can be effectively treated in non-Hodgkin lymphomas (42). Consistently, Dox causes intracellular reactive oxygen species (ROS) accumulation in cardiomyocytes, disrupted mitochondria, and triggers cardiomyocyte apoptosis via the AMPK/mTOR pathway (43) and induces apoptosis of the soleus muscle through the Akt-mTOR pathway (44). In contrast, the reduced expression of Wnt/β-catenin and PI3K/AKT/mTOR pathways induces apoptosis of T- ALL cells (45). Clearly, the regulation of Dox on function of the above-mentioned cells has not yet been investigated through A20 expression levels. Recently, toll-like receptor (TLR)-2 and TLR-9 are reported to involve in regulating Dox-induced acute inflammation and cell death in mouse models (31). Unlike Dox, Vinc activates p38 MAPK to induce the release of IL-1β in macrophages (26). 

In addition, we observed the promoting effect of Dox, but not Vinc on phagocytosis of lymphoma cells by macrophages, as the increased levels of CD68^+^CFSE^+^ cells were detected in the presence of Dox. The capacity of macrophages in phagocyting lymphoma cells is indicated in aggressive B cell lymphomas (46). In this study, LPS induced the uptake of lymphoma cells by macrophages in healthy controls, but not in cHL patients, indicating that phagocytic function of macrophages in these patients was altered. Moreover, the phagocytosis of macrophages in cHL patients was increased in the presence of Dox, but not Vinc. In agreement, the phagocytosis of cancer cells by antigen-presenting cells, including macrophages is enhanced when exposed to Dox (27). However, the regulatory effects of *A20* and signaling mechanisms underlying the uptake of lymphoma cells by macrophages was not observed in this study.

**Table 1:** List of primers used for qPCR

Gene	Forward (5’→3’)	Reverse (5’→3’)
*A20*	TCCTCAGGCTTTGTATTTGA	TGTGTATCGGTGCATGGTTTT
*CYLD*	TGCCTTCCAACTCTCGTCTTG	AATCCGCTCTTCCCAGTAGG
*mTOR*	TTCCGACCTTCTGCCTTCAC	CCACAGAAAGTAGCCCCAGG
*MAPKp38*	ATGCCGAAGATGAACTTTGC	TCTTATCTGAGTCCAATACAAGCATC
*ERK2*	CCCAAATGCTGACTCCAAAGC	GCTCGTCACTCGGGTCGTAAT
*Wnt1*	ATCTTCGCTATCACCTCCGC	GGCCGAAGTCAATGTTGTCG
*Wnt3*	TGACTCGCATCATAAGGGGC	GCCTCGTTGTTGTGCTTGTT
*AKT1*	CAGGATGTGGACCAACGTGA	AAGGTGCGTTCGATGACAGT
*Bax*	TGGCAGCTGACATGTTTT CTGAC	TCACCCAACCACCCTGGTCTT
*Caspase 3*	TGCATACTCCACAGCACCTGGTTA	CATGGCACAAAGCGACTGGATGAA
*Caspase 8*	CTGCTGGGGATGGCCACTGTG	TCGCCTCGAGGACATCGCTCTC
*GAPDH*	GGAGCGAGATCCCTCCAAA	GGCTGTTGTCATACTTCTCAT

**Table 2:** Association between A20/CYLD gene expression and clinical features in cHL patients

Characteristics	Normal range	A20 expression levels			CYLD expression levels		
		Low (n=40)	High (n=18)	*P-*value	Low (n=44)	High (n=14)	*P-*value
Age (years)		35.03 (16-70)	36.56 (16-76)	0.754	34.07 (13-76)	39.21 (20-60)	0.307
Sex, Female (n, %)		18 (45)	8 (44.44)	0.87	20 (45.5)	5 (35.7)	0.585
Urea (mmol/l)	3.3-6.6	4.69±1.55	4.65±1.06	0.914	4.7±1.47	4.61±1.2	0.825
Glucose (mmol/l)	3.9-5.6	6.17±7.62	4.91±1.07	0.492	6.05±7.22	4.84±0.72	0.539
Creatinine ( µMol/l)	50-110	71.35±18.6	75.85±13.03	0.357	69.84±16.2	82.09±16.58	0.017*
Uric acid ( µMol/l)	< 420	331.16±79.88	307.53±81.84	0.317	319.3±85	350.7±67.8	0.228
Total bilirubin ( µMol/l)	0-21	9.92±6.2	16.74±34.32	0.225	12.72±22.3	9.4±3.22	0.587
Direct bilirubin ( µMol/l)	0-7	2.99±2.52	7.64±22.44	0.263	5.13±14.7	2.48±2.04	0.576
Indirect bilirubin ( µMol/l)	1-17	7.6±2.59	10.77±15.84	0.382	9.43±11.65	7.17±2.46	0.594
Total protein (g/l)	60-80	81.28±7.85	75.94±8.1	0.035*	78.01±7.05	83.43±10.32	0.049*
Albumin (g/l)	35-52	39.19±5.84	38.33±4.26	0.585	38.92±5.36	39.22±5.56	0.863
Globulin (g/l)	20-35	42.82±10.14	38.69±8.66	0.236	39.6±7.66	46.17±13.7	0.094
Ferritin (ng/ml)	10-300	799.64±689.9	678.53±788.8	0.648	789.97±750.76	627.96±611.19	0.604
AST (GOT) (U/l)	5-40	23.39±15.64	21.56±13.74	0.285	25.84±16.34	22.23±9.2	0.451
ALT (GPT) (U/l)	7-55	41.94±39.3	18.89±12.7	0.025*	37.77±36.98	26.55±27.4	0.315
LDH (U/l)	0-247	325.67±219.32	377.2±207.49	0.464	339.83±209.58	343.05±237.9	0.965
β2 microglobulin (mg/l)	0.8-2.4	2.79±2.14	2.43±1.25	0.56	2.63±1.69	2.94±2.51	0.636
Erythrocytes (×10*12 cells/l)	4.2-5.9	4.59±0.78	4.6±0.84	0.969	4.57±0.86	4.67±0.48	0.672
Hemoglobin (g/l)	130-180	125.12±25.85	123.61±20.59	0.827	123.17±26.21	130.21±14.14	0.341
Hematocrit (%)	42-52	39±6	38,7±6	0.785	39±6	40±4	0.493
WBC count (×10*9/l)	5–14.5	12.12±5.55	12.12±5.67	0.998	11.71±5.56	13.33±5.29	0.951
Neutrophil count (×10*9/l)	1.6-7.5	8.55±5.04	9.25±5.11	0.625	8.38±4.93	9.95±5.19	0.306
Lymphocyte count (× 10*9/l)	0.9-3.4	2.06±2.31	1.6±1.03	0.43	1.95±2.21	1.8±1	0.807
Monocyte count (×10*9/l)	0-1.2	0.77±0.36	0.69±0.35	0.444	0.74±0.38	0.79±0.27	0.654
Eosinophil count (×10*9/l)	0-0.8	0.26±0.24	0.34±0.25	0.27	0.28±0.27	0.283±0.16	0.981
Basophil count (×10*9/l)	0-0.3	0.07±0.09	0.08±0.07	0.713	0.07±0.065	0.11±0.12	0.087
Platelet count (×10*9/l)	150–400	353.46±122.9	348.46±92.33	0.888	349.39±113.83	351.57±118.66	0.951

ALT: alanine aminotransferase; AST: aspartate transaminase; BM: Bone marrow; GGT: gamma glutamyl transferase; LDH: lactate dehydrogenase; WBC: white blood cell; cHL: classical hodgkin lymphoma. Statistically significant results were represented in bold style.

A, D. The dot plots of CD68^+^CD40^+^, CD68^+^CD86^+ ^(A), and Annexin V^-^PI^+^ (D) cells, which were supplemented with Vinc (150nM) or Dox (5 µM). B-C and E-G. Graphs indicate numbers of CD68^+^CD40^+ ^and CD68^+^CD86^+^ cells (B), IL-1β concentration (C), the number of Annexin V^-^PI^+^ cells (E) and mRNA levels of Bax, caspase 3 (F) and mTOR (G) in macrophages, which were untreated or treated with Vinc (150 nM) or Dox (5 µM). * (*P*<0.05) and ** (*P*<0.01) show significant differences from control cells (Mann–Whitney U test). cHL: classical hodgkin lymphoma

A, E. Original dot plots of CD68+CD40+ (A) and Annexin V-PI+ (E) cells, which were added with control siRNA or A20 siRNA or CYLD siRNA in combination with Dox (5 µM). B-D and F-G. Graphs indicate percentages of CD68+CD40+ cells (B), IL-1β concentration (C), the percentage of Annexin V-PI+ cells (D) and mRNA levels of Bax, caspase 3 (F) and mTOR (G) in macrophages, which were added with control siRNA or A20 siRNA or CYLD siRNA in combination with Dox (5 µM). * (*P*<0.05) and ** (*P*<0.01) show significant level differences from control cells. # (*P*<0.05) and ## (*P*<0.01) show significant level differences from control siRNA-treated cells (Mann–Whitney U test).

cHL: classical hodgkin lymphoma

A, D. Original dot plots of CD68+CD40+ (A) and Annexin V-PI+ (D) cells, which were added with control siRNA or A20 siRNA in combination with Dox (5 µM) or Everolimus (10 µM). B-C and E. Graphs indicate percentages of CD68+CD40+ (B) and Annexin V-PI+ cells (C) and mRNA levels of caspase 3 (E) in macrophages, which were added with control siRNA or A20 siRNA in combination with Dox (5 µM) or Everolimus (10 µM). * (*P*<0.05) shows a significant level difference from A20 siRNA-treated cells (Mann–Whitney U test). cHL: classical hodgkin lymphoma

A. Original dot plots of CD68+CFSE+ cells from healthy controls and cHL patients, which were stimulated with LPS (1 µg/ml). B. Graph indicates percentages of CD68+CFSE+ cells from healthy controls and cHL patients, which were stimulated with LPS (1µg/ml). C. Graph indicates percentages of CD68+CFSE+ cells from cHL patients, which were untreated or treated with Vinc (150nM) or Dox (5 µM). D. Original dot plots of CD68+CFSE+ cells from cHL patients, which were untreated or treated with Vinc (150 nM) or Dox (5 µM). * (*P*<0.05) shows significant difference from control cells, ### (*P*<0.01) shows significant difference from healthy individuals (Mann–Whitney U test). cHL: classical hodgkin lymphoma

## Conclusion

The present study discloses that *A20* inhibits Dox-induced macrophage maturation and apoptosis through mTOR signaling. Activation of macrophages upon treatment of Dox in HL is potentially fatal. Therefore, inhibition of Dox-induced macrophage maturation in the patients with the low A20 expression by mTOR inhibitors Everolimus may represent a promising therapy for cHL. The results may be linked to the effect of Dox on eliminating cancer cells in *A20*-sensitive cHL cases. 

## Declaration

We have not used any AI tools or technologies to prepare this manuscript.

## Limitation

There are several potential limitations in current study. The number of MCSF-diferentiated-macrophages is not large enough for analyzing protein expression of the signaling molecules. Besides, further experiments on other immune and HRS cells are necessary for investigating the differentiation/maturation, inflammatory response and cell death, as well as the molecular mechanisms underlying the regulation of *A20/CYLD* on function of Dox-treated cells in cHL patients.
